# Topological Junction States in Graphene Nanoribbons: A Route to Topological Chemistry

**DOI:** 10.1021/acs.nanolett.5c02355

**Published:** 2025-06-18

**Authors:** Hazem Abdelsalam, Domenico Corona, Renebeth B. Payod, Mahmoud A. S. Sakr, Omar H. Abd-Elkader, Qinfang Zhang, Vasil A. Saroka

**Affiliations:** † School of Materials Science and Engineering, 74619 Yancheng Institute of Technology, Yancheng 224051, P.R. China; ‡ Theoretical Physics Department, 9318 National Research Centre, Giza, 12622 Dokki, Egypt; ¶ Department of Physics, 54729 University of Rome Tor Vergata and INFN, Via della Ricerca Scientifica 1, 00133 Roma, Italy; § 110121 Institute of Physics, University of the Philippines, College, Los Baños, Laguna 4031, Philippines; ∥ Chemistry Department, Center of Basic Science, 37850 Misr University for Science and Technology (MUST), P.O. Box 77, Giza 12566, Egypt; ⊥ Department of Physics and Astronomy, College of Science, King Saud University, P.O. Box 2455, Riyadh 11451, Saudi Arabia; # 8018 TBpack Ltd., 27 Old Gloucester Street, London WC1N 3AX, United Kingdom; @ Institute for Nuclear Problems, Belarusian State University, Bobruiskaya 11, 220030 Minsk, Belarus

**Keywords:** graphene, topological phases, density functional theory, tight-binding model, quantum transport, gas sensing

## Abstract

Two-dimensional topological insulators with propagating topological edge states are promising for dissipationless transport, while their one-dimensional analogs are capable of hosting localized topological junction states that are mainly envisaged for quantum computing and spintronics. Here, in contrast, we propose to use the localized nature of topological junction states for sensing applications. We report a systematic topological classification of a wide class of graphene nanoribbons represented by already synthesized extended chevron species. Using this classification, we theoretically model a double junction transport that shows an enhanced interaction with the NO_2_ molecule. Our results show that topological junction states of nanoribbons can open an avenue for topological sensing and junction-assisted chemistry applications.

## Graphical abstract

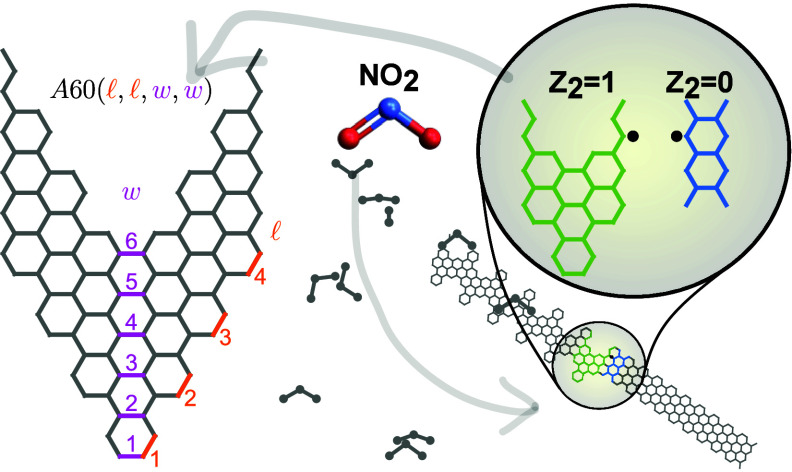

The exploration of topological states in condensed matter physics has revolutionized our understanding of electronic materials,^1–3^ opening new pathways for advanced technological applications in electronics and error-resilient quantum information.^4^ Among the myriads of topologically intriguing systems,^5–7^ carbon-based nanostructures played a key role from the beginning.^8,9^ In recent years, particularly graphene nanoribbons (GNRs) have garnered significant attention.^10–22^ These one-dimensional (1D) nanostructures, derived from graphene, exhibit unique electronic properties due to their distinct edge terminations and narrow widths.^23,24^

The bulk-boundary correspondence principle is at the heart of the topological band theory of solids. It states that, at the interfaces between regions of different topological orders, topological interface states occur as highly localized electronic modes that are resilient to disorder or perturbations.^1^ The topological stability of these interface states can be controlled by several topological invariants. In the case of the integer quantum Hall effect^25,26^ or Haldane model,^27^ the invariant is the Chern number, while it is *Z*_2_ invariant instead for topological insulators.^8,9^ The *Z*_2_ invariant provides the binary classification of topological phases with values of 0 or 1. A wider classification based on the Chern number utilizes the whole set of integer numbers *Z*. In both cases, a zero value stands for a trivial topological order. An interface state appears, whenever two regions with different *Z* or *Z*_2_ values meet each other. This basic principle works in all dimensions and has recently attracted a lot of attention for 1D structures, such as graphene nanoribbons, where topological junction states (TJS) have been revealed and classified.^10,11,17,18^ The classification of TJS in GNRs can be carried out based on *Z*_2_ invariant^10–12^ and also can be generalized by incorporating chiral symmetry and spin corrections.^17^ Some of these states have already been experimentally shown and investigated through an atomically precise on-surface bottom-up engineering.^14,15,21,28^

The envisaged applications of topologically protected states are (i) disorder-resilient low-dissipation transport and (ii) robust long-coherence spin states.^2,4^ The latter has become a roadmap for topological junction states as potential candidates for designing customizable spintronics^10,13,20^ and quantum computing^13,20^ nanodevices. Notably, this widely accepted roadmap overlooks chemical sensing, which is arguably a more natural and immediate application. Chemical sensing relies critically on the interaction between the sensor material and the target analyte, where sensitivity and selectivity are paramount.^29^ The 1D nature of GNRs, combined with topological junctions providing a high density of localized electronic states to facilitate interaction with chemical species, potentially offers enhanced sensitivity and selectivity that are accessible for readout in a standard transport device similar to a field-effect transistor.^30,31^

This paper presents a theoretical investigation of topological junction states in GNRs, with a particular focus on their application in gas sensing. The gas sensing application has already been proposed and investigated both theoretically^32–35^ and experimentally^36,37^ for the edge and end states of GNRs; see also a review of more general sensing schemes in ref 31. Some experimental studies have demonstrated the detection of volatile compounds such as ethanol and methanol^36^ as well as nitrogen dioxide NO_2_.^37^ However, the sensitivity of TJS in GNRs remains unknown. Here, we explore the electronic properties of these states, their formation mechanisms, and their interaction with NO_2_ as the target molecule. We have noticed that some of the ribbons that we have studied previously^34^ (we refer to those as A60 within a more general class of zigzag-shaped GNRs^38–41^ ), are, in fact, topologically nontrivial. For some combinations of their unit cell structural parameters, they are characterized by the *Z*_2_ invariant derived from the intercellular Zak phase^42,43^ calculated in the periodic gauge of the tight-binding (TB) Hamiltonian.^3^ Thus, they can form TJSs via seamless combination with a topologically trivial unit cells of armchair GNRs (AGNRs).^10^ Performing density functional theory (DFT) calculations to study sensing, we focus on nitrogen dioxide (NO_2_) gas, which is a free-radical and a moderate Lewis acid. NO_2_ is an air pollutant affecting the environment and health of people^44,45^ and a prototype of a vast range of substances belonging to nitrocompound group, e.g. nitroparaffins and nitroarenes. By leveraging the unique properties of topological junctions in GNRs, we aim to elucidate their potential in creating highly sensitive and selective chemical sensors, paving the way for advancements in environmental monitoring, and industrial safety.^46^

## Topological Properties

The TB Hamiltonian of π-electron network is (neglecting a vanishing spin–orbit term^47^)­

1 $$H=\underset{i}{\sum }\varepsilon c_{i}^{†}c_{i}+\underset{i,j}{\sum }t_{1}\left(c_{i}^{†}c_{j}+c_{j}^{†}c_{i}\right)$$

where *c*_*i*_^†^ and *c*_*i*_ are electron creation and annihilation operators, respectively, *ε* = 0 is the on-site energy equal for all lattice sites, *t*_1_ = 3.12 eV^48^ is the nearest-neighbor hopping integral. We note here that *t*_1_ varies for different structures,^49,50^ therefore, we will use *E*/*t*_1_ dimensionless units in what follows. By going into reciprocal *k*-space in eq 1 via a Fourier transform, $c_{i}^{†}=\frac{1}{\sqrt{N}}\underset{k}{\sum }e^{-i{\mathrm{kR}}_{i}}c_{k}^{†}$ (and similar for *c*_*i*_) with *N* being the number of unit cells and *R*_*i*_ being the site position, we solve the eigenproblem of *n* × *n* matrix Hamiltonian for a given unit cell of a GNR with *n* lattice sites in the cell, using *TBpack* package.^51,52^ Then *k*-space Hamiltonian eigenvectors, *C*_*i*_(*k*) = (*C*_*i*,1_(*k*), *C*_*i*,2_(*k*), ..., *C*_*i,n*_(*k*))^T^, can be employed for the calculation of the intercellular Zak phase by eq 31 in ref 42

2 γ2=−Im{log⁡∏j=1NkDet[Cp†(kj)Cq(kj+1)]}

where *N*_*k*_ is the number of *k*-point sampling the Brillouin zone of the GNR, and the indices *p* and *q* run from 1 to *n*/2 so that dot products of eigenvectors in eq 2 form a matrix from all occupied states.

In Table 1, six topological junctions of interest are constructed. The first two cases are dealing with chevron-type GNR (cGNR)^54^ and AGNRs, classified by the number of carbon atom pairs in their unit cells *N*_*p*_, i.e. AGNR­(*N*_*p*_). Those Cases 1 and 2 are used to verify topological band theory in finite-size cluster approach to supplement periodic calculations of refs 10 and 11 . Cases 3 and 4 present the topological junctions in focus of the given study, constructed using the A60 class of GNRs^38,39,41^ defined by a set of four structural parameters (*l*_1_, *l*_2_; *w*_1_, *w*_2_), that are explained in Scheme 1.

**Table 1:**
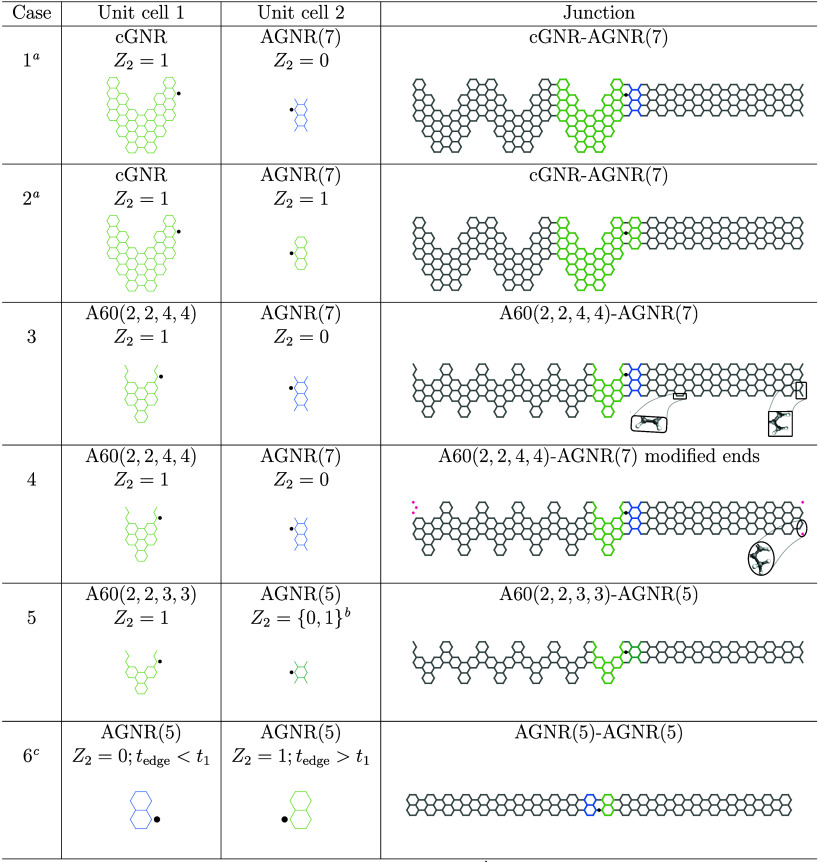
Junctions between Various Types of GNRs^a^

a Junctions previously reported, see Figure 2b in ref 11.

b Ambiguity of *Z*_2_ with respect to applied strain has been reported in ref 53; see, for example, Figure 2c. Here the structure is considered to be gapless, i.e. metallic.

c This junction is obtained by the reducing model of ref 53, that accounts for up to third nearest neighbor hopping interactions.

d Light green marks topological structures, while light blue and green-blue marks are trivial and undefined, i.e. “metallic”, structures, respectively. Black dots are the alignment points for a pair of unit cells forming a junction. Crimson dots show excluded from *π*-network C atoms that are not bonded to as many C atoms as they would be in the bulk of the structure. Insets: possible chemical terminations. The period of translation for the bulk structures is along horizontal *Ox*-axis.

**Scheme 1:**
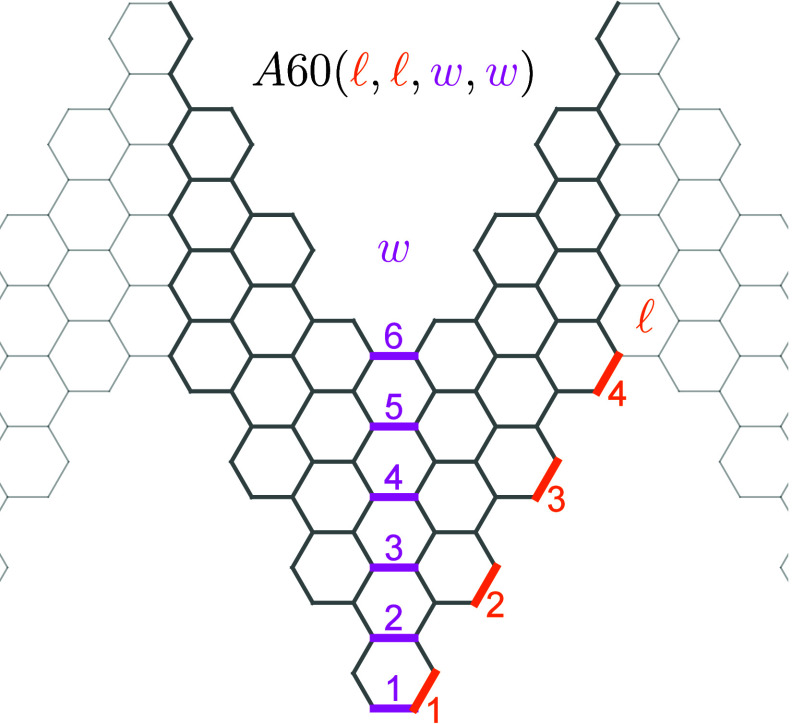
Main Structural Parameters of a Unit Cell of a Mirror Symmetric A60 Class of GNRs[Fn sch1-fn1]

a
*l*_1_ = *l*_2_ = *l* and *w*_1_ = *w*_2_ = *w*. A60­(*l*_1_,*l*_2_,*w*_1_,*w*_2_) ⇒ A60 (4,4,6,6) is taken as an example. It was synthesized in ref 36. The period of translation is along the horizontal axis.

The *Z*_2_ topological classification of symmetric A60 GNRs, *l* = *l*_1_ = *l*_2_ and *w* = *w*_1_ = *w*_2_, is summarized in Table 2. For the sake of completeness, Cases 5 and 6 dealing with metallic structures^55^ and strain^53^ are added to Table 1.

**Table 2:**
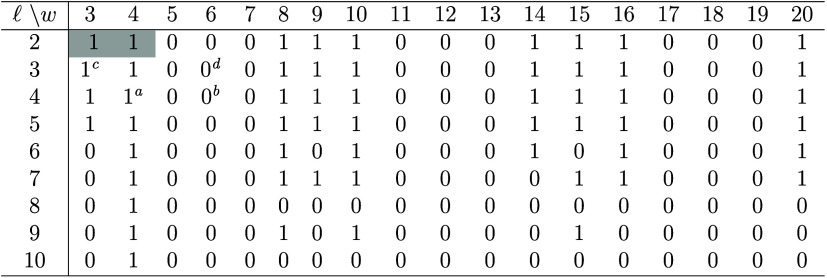
*Z*_2_ Topological Invariant of A60 (*l*_1_, *l*_2_; *w*_1_, *w*_2_) GNRs as a Function of Their Structural Parameters *l* = *l*_1_ = *l*_2_ and *w* = *w*_1_ = *w*_2_ ^a^

a Figure 2 in ref 38.

b Figure 2b in ref 11 and Figure 1 in ref 36.

c Figure 2a,d in ref 34.

d Figure 2b,e in ref 34.

e Values in light gray are for A60 GNRs used further in this study. Footnotes *a*–*d* refer to species previously studied in some aspects.

**Figure 1:**
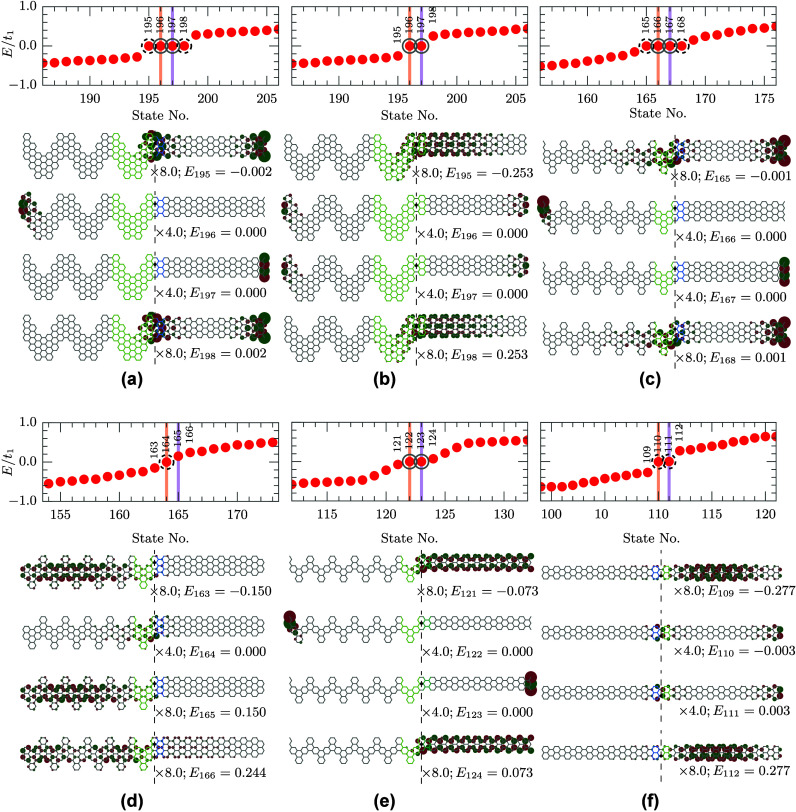
Electronic properties of topological junctions. (a) Energy levels for cGNR-AGNR(7) topological-trivial junction. Vertical orange and violet lines mark ⌈N/2⌉ and ⌈N/2⌉+1 , with ⌈x⌉ and *N* being the ceiling operation and total number of C atoms in the structure, respectively. Dashed black circles highlight TJSs, gray circles highlight end states. The wave functions of the numbered states are presented below, where the scaling factors together with the state energies in terms of *E*/*t*_1_ are given. Dark red and green show the positive and negative phase of the wave function, respectively. Vertical dashed black lines denote the junction. Black dot is the alignment point for the two underlined unit cells. Light green and light blue highlight topological and trivial unit cells, respectively. (b) Same as (a) but for cGNR-AGNR(7) topological-topological junction. (c) Same as (a) but for A60­(2, 2, 4, 4)-AGNR(7) topological-trivial junction. (d) Same as (a) but for A60­(2, 2, 4, 4)-AGNR(7) junction with modified ends. (e) Same as (a) but for A60­(2, 2, 3, 3)-AGNR(5) ill-defined junction. The green blue color highlights the “metallic” unit cell. (f) Same as (a) but for the edge-strain-induced junction in AGNR(5): *t*_edge_ = 0.5 *t*_1_ to the left and 1.5 *t*_1_ to the right of the junction.

Figure 1 summarizes the results for the nanoribbon junctions presented in Table 1 and the states observed for them. As one can see from Figure 1a, describing the finite-size structure of Case 1 in Table 1, the zero energy hosts four states, two of which, i.e. No. 195 and 198, are localized at the junction region marked with a vertical dashed black line. These states also have some weight at the trivial end of the structure. This feature is missing in periodic structure calculations and never mentioned in ref 11. The two other states, No. 196 and No. 197, are the end states. The state No. 196 is a topological end state localized at the border of the topological cGNR unit cell and the vacuum, which is trivial insulator, *Z*_2_ = 0, because it can be explained by the bulk-boundary correspondence. In contrast, the state No. 197 forms at the interface between trivial AGNR(7) unit cell (light blue color) and the trivial vacuum which must be an ordinary end state related to carbon atoms featuring unpaired electrons as we shall see later. Figure 1b shows the energy levels and the wave functions of the cGNR-AGNR(7) junction, which is Case 2 in Table 1. In this structure, there are only two zero-energy states, which are end states. These states No. 196 and 197, respectively, are both localized at the interface between a topological unit cell and a trivial vacuum. Thus, these states are predicted by the bulk-boundary correspondence principle so that they are both topological end states. For comparison, the two bulk states are also presented in Figure 1b. Analogous relations can be noticed in Figure 1c corresponding to Case 3 in Table 1. Here we show electronic energy levels and wave functions for A60­(2, 2, 4, 4)-AGNR(7) junction between topological and trivial unit cells, respectively. Similar to Figure 1a, along with the two TJSs, No. 165 and 168, having some weight at the end of the structure, there are states No. 166 and 167 localized entirely at the ends of the structure. To verify the hypothesis that they are merely related to the C atoms featuring unpaired electrons, we constructed Case 4 structure with 5 atoms removed as shown in Table 1. Figure 1d confirms that only one TJS remains after all the C atoms carrying unpaired electrons have been removed. Finally, we present the results for the controversial Case 5. We note that eq 2 applied to the AGNR(5) yielded *Z*_2_ = 1. Despite the appeal to interpret the results in Figure 1e with the bulk-boundary correspondence as in the Case 2 discussed with Figure 1b, this approach will not work for other “metallic” structures. Due to this reason, the unit cell of AGNR(5) is highlighted with a blend between light green and light blue colors. On the other hand, when the metallic structure has a well-defined gap due to the edge strain as in Case 6, then a localized TJS is clearly identifiable, as one can see from Figure 1(f). Note that to construct the strain junction the unit cell must be chosen differently; see ref 10. Table 1 for allowed choices. Thus, we have seen that topological band theory allows us to predict and reliably construct localized junction states. From a chemistry point of view, these nucleophilic regions with excess electrons due to TJSs are similar to radicals and they must be perfect reaction sites. The geometry of the junction compatible with a standard transport experiment and radical-like nature of the TJS must make them useful for sensing application.

## Topological Sensing

The above presented junctions are all asymmetric. The asymmetry of the junctions may pose a significant problem for potential transport measurements by requiring differing fabrication approaches of the left and right leads. This problem could be bypassed in a mirror symmetric double junction (DJ). As shown in Figure 2, a DJ has a few topological unit cells of an A60 GNR sandwiched between trivial AGNRs.

**Figure 2:**
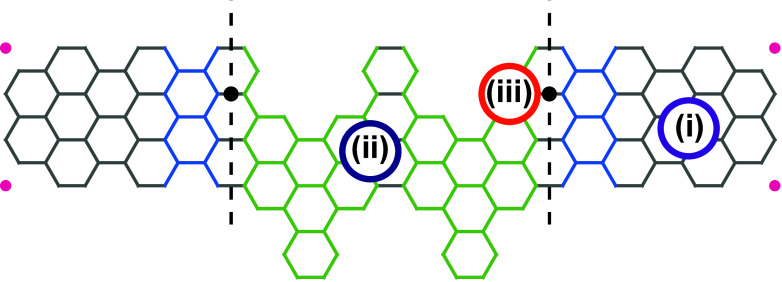
Double junction scheme. Following notation used in Table 1, the light green unit cells of A60­(2, 2, 4, 4) are topological ones, while light blue unit cells of AGNR(7) are trivial. The alignment points for the two junctions are depicted with black dots, while the junctions are marked with two dashed black lines. The C atoms that are excluded from π-electron network by their conversion into methyl groups are shown as crimson dots. The three circles highlight the chosen site for investigating adsorption properties: (i) violet - AGNR(7), (ii) dark blue - A60­(2, 2, 4, 4), and (iii) orange - one of the two topological junctions. Hydrogen atoms are not displayed.

Using DFT calculations as implemented in Gaussian 16 ^56^ and postprocessing with *Multiwfn* software^57^ for partial density of states (PDOS) extraction, we scrutinized the electronic and adsorption properties of the DJ.^52^ See further details in the Supporting Information Note 1. In the constructed double junction, the C atoms with unpaired electrons are electronically saturated via hydrogenation to form methyl groups, therefore, we expect one TJS per junction similar to Figure 1d. Since two junctions are placed close to each other in our DJ, the two TJS can slightly overlap. This interaction between TJSs shall result in their symmetric splitting around the Fermi energy, *E*_f_ = 0 eV, similar to what happens with the ground state of a double quantum well.^58^

As one can see from Figure 3(a), the DFT modeling confirms the expected ground state splitting. The DJ highest occupied molecular orbital (HOMO) and lowest unoccupied molecular orbital (LUMO) energies are separated by the gap *E*_g_ = 1.08 eV. The distributions of the HOMO and LUMO are clearly different from those of the bulk orbitals. While bulk states extend over a large number of trivial AGNR(7) unit cells, the HOMO and LUMO representing TJSs are localized at the two A60­(2, 2, 4, 4) unit cells in the region between the two junctions. These topological states are expected to be more interactive than the bulk ones. To compare TJSs to other energy states in the DJ, the reactivity is tested by studying the adsorption properties of the NO_2_ gas molecule on different sites of the DJ. In this testing, the NO_2_ is initially positioned at about 3 Å above the DJ at three sites: (i) the AGNR, (ii) the A60 GNR, and (iii) the junction. Then the whole structure is optimized so that NO_2_ ends up at the sites illustrated in Figure 2. The three chosen adsorption configurations cover reduced electron densities away from the junction and increased electron density due to TJS in the junction region. The adsorption energies in the first two cases are comparable, namely – 0.27 and – 0.30 eV, respectively. In contrast, the adsorption energy at the junction site is greater than double of these values, i.e. *E*_a_ = – 0.66 eV. This indicates that TJSs indeed boost interaction with the target molecule. Adsorption of NO_2_ is accompanied by the electron charge transfer of – 0.55*e* Mulliken charge [−0.43*e* Hirshfeld charge] from the junction to NO_2_ molecule. Before the adsorption, the O atom charge in NO_2_ molecule is – 0.20*e* [−0.1035*e*] for both oxygen atoms, while N atom charge is 0.40*e* [0.207*e*]. However, these charges decrease to – 0.42*e* [−0.247*e*] and – 0.35*e* [−0.236*e*] for oxygen atoms and to 0.22*e* [0.053*e*] for the nitrogen atom after adsorption at the junction site. For comparison, adsorption of NO_2_ on the A60 and AGNR sites results in – 0.19*e* [−0.155*e*] and – 0.11*e* [−0.081*e*] Mulliken [Hirshfeld] charge transfers, respectively. Therefore, physical adsorption at the junction site is stronger than on the other two sites. Figure 3(b) shows spin-polarized energy levels for the system, wherein NO_2_ is adsorbed on the junction spot of the DJ. The main effect of the NO_2_ adsorption is induced spin-splitting of the TJSs, both HOMO and LUMO, and the resulting band gap reduction for spin-down component: *E*_g,↓_ = 0.51 eV, as compared to *E*_g,↑_ = 1.04 eV and *E*_g_ = 1.08 eV of a spin-unpolarized case in Figure 3(a). As seen from the HOMO spin-down component, the contribution of NO_2_ into the spin-down component of the bonding HOMO is significant in the case of adsorption at the junction which is compelling evidence of the localized TJSs being more reactive than extended bulk states. The partial densities of states plotted in Figure 3(c) and (d) further demonstrate that NO_2_ contributions (dark blue and violet) into HOMOs are almost vanishing when the adsorption takes place at A60­(2, 2, 4, 4) and AGNR(7) sites even though the energy gaps are comparable to the junction case: *E*_g,↓_ = 0.71 and 0.38 eV and *E*_g,↑_ = 1.07 and 1.09 eV, respectively. This indicates that TJSs may be suitable for facilitating sensing and catalytic applications.

**Figure 3:**
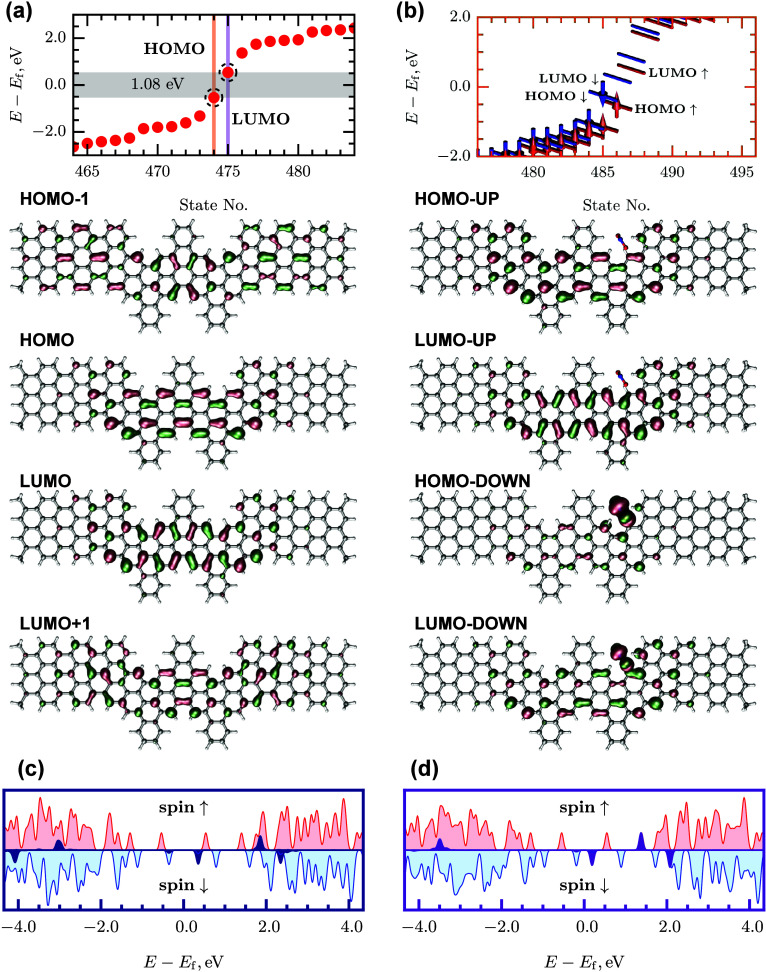
Interaction with NO_2_ gas molecule. (a) The electronic energy levels of pristine DJ together with molecular orbitals of TJS and extended bulk states. Isovalue: 0.02. Similar to Figure 1 vertical orange and violet lines mark HOMO and LUMO, while dashed black circles highlight TJSs. (b) The spin-polarized electronic energy levels for DJ after adsorption of NO_2_ molecule at the A60­(2, 2, 4, 4)-AGNR(7) junction between topological and trivial unit cells. (c) The spin-resolved partial density of states for DJ and NO_2_ molecule adsorbed at A60­(2, 2, 4, 4). The NO_2_ data are plotted by the same dark blue color that is used to denote NO_2_ adsoption site in Figure 2. (d) Same as (c), but for NO_2_ adsorbed at AGNR(7). Broadening: 0.1 eV. The frame colors in (b), (c), and (d) correspond to those of circles showing the adsoption sites in Figure 2.

## Sensor Readout with Coherent Transport

The constructed symmetric DJ has a favorable configuration for connecting leads to and passing a coherent current through it. This current must be sensitive to the electronic properties of the scattering region represented by two junctions between topological and trivial nanoribbon unit cells. The changing current shall be a signature of the physical adsorption happening at the junction. The geometry of the leads is crucial here. In order to facilitate electron injection into the semiconducting structure, we use a combination of topological and chemical engineering. Specifically, we terminate the scattering region with topologically nontrivial AGNR(7) unit cell; this engineering is equivalent to that of Figure 3 of ref 11 or the one in the recent experiment on Janus GNRs.^59^ In addition, we *n*-dope the left and right leads with N atoms as shown in Figure 4a to make them conducting; see Supporting Information Note 2 and Figures S1 and S2. The latter may be a bit challenging step from a technological point of view, though atomically precise doping of GNRs has been demonstrated^60–64^ and N–C interfaces have already been studied experimentally.^65^

**Figure 4:**
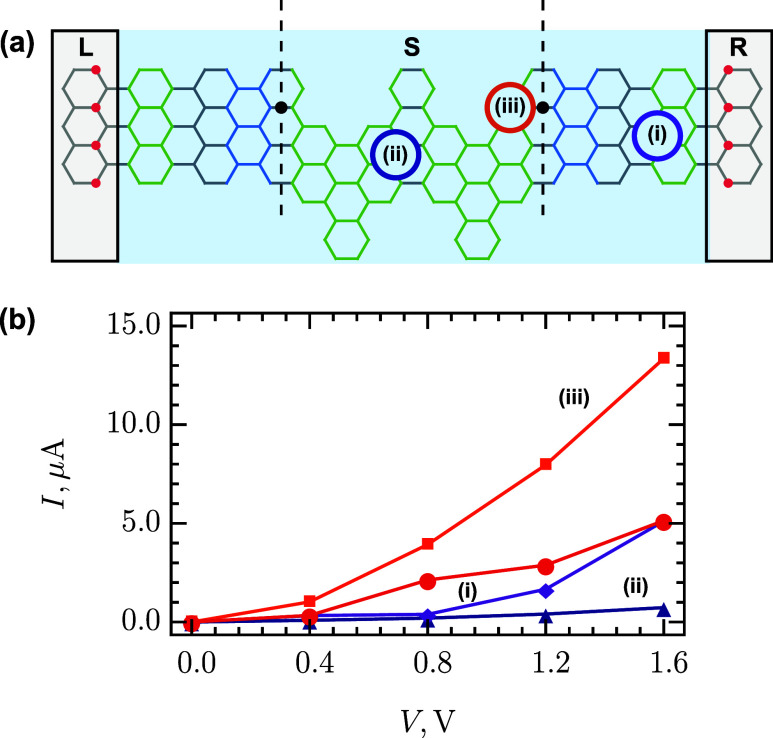
Quantum transport readout of gas molecules. (a) The scheme of the quantum transport sensor. Notations are the same as in Table 1 and Figure 2. The red dots denote nitrogen atoms. “L”, “S”, and “R” are the left lead, scattering region (whole light blue area), and right lead, respectively. (b) The current–voltage characteristics of the sensor before and after NO_2_ adsorption: (i) at AGNR(7), (ii) at A60­(2, 2, 4, 4), and (iii) the topological junction between A60­(2, 2, 4, 4) and AGNR(7) unit cells. The red [disks] are data for pristine sensor, while violet [rhombuses], dark blue [triangles] and orange [squares] are data for the sites that are marked with the same colors in panel (a).

The quantum transport through the lead-DJ-lead system, where leads are semi-infinite, is explored using DFT and nonequilibrium Green’s function methods implemented in *NanoDCAL* software,^66,67^ see ref 52 and Supporting Information Note 1. Working in a low-bias regime and accounting for well-matched lead-DJ interface with negligible relaxation and charge transfer, the nonself-consistent transport modeling is sufficient.^68^

In Figure 4b, the current–voltage (*I*-*V*) characteristics for the DJ-based sensor are presented within a reasonable range of voltages from 0 to 1.6 V. The reverse bias must have similar effect because of the mirror symmetry of the DJ, while the negative bias for the leads is not recommended due to the *n*-doping of the leads and the band gap below the Fermi level. The modeled sensor demonstrates measurable currents of about tens of μA’s. Due to not very long the AGNR(7) and A60­(2, 2, 4, 4) fragments and the lead engineering, the scattering region passes enough current. Longer segments of AGNR(7) show lower conductance in our quantum transport simulations. The adsorption of NO_2_ at the favorable junction site results in a 2-fold increase in current. Simultaneously, the NO_2_ adsorption at energetically less favorable sites of AGNR(7) and A60­(2, 2, 4, 4) blocks the current through the sensor. The presented detection mechanism differs from that of the previously proposed junction-free AGNR-based sensors.^69^ In our case, the adsorption of the molecule at the junction increases the current, whereas in other schemes the current is usually blocked.

Finally, we estimate the recovery time to obtain critical insights into NO_2_ desorption dynamics of the constructed sensor and to reveal its effectiveness and long-term applicability.^52^ The recovery time (τ) is calculated using the transition state theory Arrhenius-type equation^70,71^

3 $$\tau =\nu^{-1}\;\exp\left(-\frac{E_{\mathrm{ac}}}{k_{B}T}\right)$$

where ν is the attempt frequency, *E*_a_ is the activation energy, *k*_B_ is Boltzmann’s constant in eV/K and *T* is the temperature in K. The value of the attempt frequency is taken to be 10^12^ s^–1^ as suggested for carbon nanotube-based NO_2_ sensor in ref 72. At the room temperature *T* = 298 K, the values of τ for adsorption sites (i), (ii) and (iii) are 3.68 × 10^–8^, 1.19 × 10^–7^, and 0.15 s, respectively. The recovery time for NO_2_ adsorption at the topological junction site (iii) is significantly longer than at the AGNR or A60 sites which means the sensor could be used as is, i.e. without capping these areas with protective layers. Concurrently, 0.15 s is more than 10-fold shorter than experimental 2 s recovery times recently reported for In_2_O_3_-nanoparticle-based NO_2_ sensor.^73^ This suggests that our sensor could support a high frequency of measurements and rapid data collection.

## Summary and Conclusions

We report a systematic topological classification for an A60 class of graphene nanoribbons, which can be used in a mirror symmetric double junction configuration as active elements of a gas sensor that is read out by quantum transport measurements. This envisages a niche for sensor applications of topological materials, in addition to the quantum computing perspective suggested in the literature. In view of the recent advance in an experimental study of topological end states in germanene nanoribbons,^74^ we infer that our calculations can also be extended to inorganic chemistry; or even metamaterials.^75–78^

## Supplementary Material



## Notes

Details of our electronic structure and transport calculations, results on electronic and topological properties of the N-doped sensor leads modeled at the tight-binding and density functional theory levels (PDF)

## References

[1] Hasan M. Z., Kane C. L. (2010). Colloquium: Topological insulators. Rev. Mod. Phys..

[2] Ren Y., Qiao Z., Niu Q. (2016). Topological phases in two-dimensional
materials: a review. Rep. Prog. Phys..

[3] Cayssol J., Fuchs J. N. (2021). Topological and
geometrical aspects of band theory. J. Phys.
Mater..

[4] Weber B. (2024). 2024 Roadmap on 2D Topological
Insulators. J. Phys. Mater..

[5] Qi X.-L. L., Zhang S.-C. C. (2011). Topological insulators and superconductors. Rev. Mod. Phys..

[6] Ando Y. (2013). Topological
insulator materials. J. Phys. Soc. Jpn..

[7] Wehling T., Black-Schaffer A., Balatsky A. (2014). Dirac materials. Adv. Phys..

[8] Kane C. L., Mele E. J. (2005). Quantum spin Hall
effect in graphene. Phys. Rev. Lett..

[9] Kane C. L., Mele E. J. (2005). Z_2_ topological
order and the quantum spin
Hall effect. Phys. Rev. Lett..

[10] Cao T., Zhao F., Louie S. G. (2017). Topological
phases in graphene nanoribbons:
Junction States, spin centers, and quantum spin shains. Phys. Rev. Lett..

[11] Lee Y.-l., Zhao F., Cao T., Ihm J., Louie S. G. (2018). Topological
phases in cove-edged and chevron graphene nanoribbons: Geometric structures,
Z_2_ invariants, and junction states. Nano Lett..

[12] Lin K.-S., Chou M.-Y. (2018). Topological
properties of gapped graphene nanoribbons
with spatial symmetries. Nano Lett..

[13] Ortiz R., García-Martínez N. A., Lado J. L., Fernández-Rossier J. (2018). Electrical
spin manipulation in graphene nanostructures. Phys. Rev. B.

[14] Rizzo D. J., Veber G., Cao T., Bronner C., Chen T., Zhao F., Rodriguez H., Louie S. G., Crommie M. F., Fischer F. R. (2018). Topological band
engineering of graphene nanoribbons. Nature.

[15] Gröning O., Wang S., Yao X., Pignedoli C. A., Borin Barin G., Daniels C., Cupo A., Meunier V., Feng X., Narita A., Müllen K., Ruffieux P., Fasel R. (2018). Engineering of robust topological
quantum phases in graphene nanoribbons. Nature.

[16] Joost J. P., Jauho A. P., Bonitz M. (2019). Correlated
topological states in
graphene nanoribbon heterostructures. Nano Lett..

[17] Jiang J., Louie S. G. (2021). Topology classification
using chiral symmetry and spin
correlations in graphene nanoribbons. Nano Lett..

[18] Arnold F. M., Liu T.-J., Kuc A., Heine T. (2022). Structure-imposed electronic
topology in cove-edged graphene nanoribbons. Phys. Rev. Lett..

[19] Kuo D. M. T. (2023). Effects
of coulomb blockade on the charge transport through the topological
states of finite armchair graphene nanoribbons and heterostructures. Nanomaterials.

[20] Perkins D. T. S., Ferreira A. (2024). Ultrafast all-electrical universal nanoqubits. Phys. Rev. B.

[21] Wang Z., Yin R., Tang Z., Du H., Liang Y., Wang X., Deng Q.-S., Tan Y.-Z., Zhang Y., Ma C., Tan S., Wang B. (2024). Topologically localized vibronic excitations in second-layer
graphene nanoribbons. Phys. Rev. Lett..

[22] Zhao C., Catarina G., Zhang J.-J., Henriques J. a. C. G., Yang L., Ma J., Feng X., Gröning O., Ruffieux P., Fernández-Rossier J., Fasel R. (2024). Tunable topological
phases in nanographene-based spin-1/2 alternating-exchange Heisenberg
chains. Nat. Nanotechnol..

[23] Yano Y., Mitoma N., Ito H., Itami K. (2020). A quest for
structurally
uniform graphene nanoribbons: Synthesis, properties, and applications. J. Org. Chem..

[24] Chen Z., Narita A., Müllen K. (2020). Graphene nanoribbons: On-Surface
synthesis and integration into electronic devices. Adv. Mater..

[25] Simon B. (1983). Holonomy,
the quantum adiabatic theorem, and Berry’s phase. Phys. Rev. Lett..

[26] Niu Q., Thouless D. J., Wu Y.-S. (1985). Quantized Hall conductance as a topological
invariant. Phys. Rev. B.

[27] Haldane F. D. M. (1988). Model
for a quantum Hall effect without landau levels: Condensed-matter
realization of the “parity anomaly”. Phys. Rev. Lett..

[28] Wang S., Talirz L., Pignedoli C. A., Feng X., Müllen K., Fasel R., Ruffieux P. (2016). Giant edge
state splitting at atomically
precise graphene zigzag edges. Nat. Commun..

[29] Gao N., Gao T., Yang X., Dai X., Zhou W., Zhang A., Lieber C. M. (2016). Specific detection
of biomolecules in physiological
solutions using graphene transistor biosensors. Proc. Natl. Acad. Sci. U.S.A..

[30] Adamu B. I., Chen P., Chu W. (2021). Role of nanostructuring of sensing
materials in performance of electrical gas sensors by combining with
extra strategies. Nano Ex..

[31] Johnson A. P., Sabu C., Swamy N. K., Anto A., Gangadharappa H., Pramod K. (2021). Graphene nanoribbon: An emerging
and efficient flat
molecular platform for advanced biosensing. Biosens. Bioelectron.

[32] Abdelsalam H., Saroka V. A., Younis W. O. (2019). Edge functionalization of finite
graphene nanoribbon superlattices. Superlattices
Microstruct..

[33] Čerņevičs K., Pizzochero M., Yazyev O. V. (2020). Even-odd conductance effect in graphene
nanoribbons induced by edge functionalization with aromatic molecules:
Basis for novel chemosensors. Eur. Phys. J.
Plus.

[34] Abdelsalam H., Saroka V., Teleb N., Ali M., Osman W., Zhang Q. (2021). Electronic and adsorption properties
of extended chevron and cove-edged
graphene nanoribbons. Physica E Low Dimens..

[35] Sakr M. A., Abdelsalam H., Teleb N. H., Abd-Elkader O. H., Saroka V. A., Zhang Q. (2024). Investigating
adsorption characteristics
and electronic properties of Clar’s goblet and beyond. Chem. Phys. Lett..

[36] Mehdi
Pour M., Lashkov A., Radocea A., Liu X., Sun T., Lipatov A., Korlacki R. A., Shekhirev M., Aluru N. R., Lyding J. W., Sysoev V., Sinitskii A. (2017). Laterally
extended atomically precise graphene nanoribbons with improved electrical
conductivity for efficient gas sensing. Nat.
Commun..

[37] Cho K. M., Cho S.-Y., Chong S., Koh H.-J., Kim D. W., Kim J., Jung H.-T. (2018). Edge-functionalized
graphene nanoribbon chemical sensor:
Comparison with carbon nanotube and graphene. ACS Appl. Mater. Interfaces.

[38] Saroka V. A., Batrakov K. G., Chernozatonskii L. A. (2014). Edge-modified zigzag-shaped graphene
nanoribbons: Structure and electronic properties. Phys. Solid State.

[39] Saroka V. A., Batrakov K. G., Demin V. A., Chernozatonskii L. A. (2015). Band gaps
in jagged and straight graphene nanoribbons tunable by an external
electric field. J. Phys.: Condens. Matter.

[40] Saroka, V. A. ; Batrakov, K. G. In Physics, Chemistry and Applications of Nanostructures; Borisenko, V. E. , Gaponenko, S. V. , Gurin, V. S. , Kam, C. H. , Eds.; World Scientific: 2015; pp 240–243.

[41] Saroka V. A., Batrakov K. G. (2016). Zigzag-shaped Superlattices on the basis of graphene
nanoribbons: Structure and electronic properties. Russ. Phys. J..

[42] Kudin K. N., Car R., Resta R. (2007). Berry phase approach
to longitudinal dipole moments
of infinite chains in electronic-structure methods with local basis
sets. J. Chem. Phys..

[43] Rhim J.-W., Behrends J., Bardarson J. H. (2017). Bulk-boundary correspondence from
the intercellular Zak phase. Phys. Rev. B.

[44] Last J. A., Sun W. M., Witschi H. (1994). Ozone, NO,
and NO_2_: oxidant
air pollutants and more. Environ. Health Perspect..

[45] Huangfu P., Atkinson R. (2020). Long-term exposure
to NO_2_ and O_3_ and all-cause and respiratory
mortality: A systematic review and
meta-analysis. Environ. Int..

[46] Li Q., Zeng W., Li Y. (2022). Metal oxide gas sensors for detecting
NO_2_ in industrial exhaust gas: Recent developments. Sens. Actuators B: Chem..

[47] Sichau J., Prada M., Anlauf T., Lyon T. J., Bosnjak B., Tiemann L., Blick R. H. (2019). Resonance microwave
measurements
of an intrinsic spin-orbit coupling gap in graphene: A possible indication
of a topological state. Phys. Rev. Lett..

[48] Partoens B., Peeters F. M. (2006). From graphene to
graphite: Electronic structure around
the K point. Phys. Rev. B.

[49] Saroka V. A., Abdelsalam H., Demin V. A., Grassano D., Kuten S. A., Pushkarchuk A., Pulci O. (2018). Absorption in finite-length
chevron-type
graphene nanoribbons. Semiconductors.

[50] Payod R. B., Grassano D., Santos G. N. C., Levshov D. I., Pulci O., Saroka V. A. (2020). 2N+4-rule and an
atlas of bulk optical resonances of
zigzag graphene nanoribbons. Nat. Commun..

[51] Saroka, V. A. TBpack (version 0.5.2 and higher). 2025; https://github.com/vasilsaroka/TBpack, Accessed 2025-04-25.

[52] See Supplementary Materials at 10.5281/zenodo.15209274 that include TB and DFT calculation raw data and Mathematica code to reproduce the reported results.

[53] Tepliakov N. V., Lischner J., Kaxiras E., Mostofi A. A., Pizzochero M. (2023). Unveiling
and manipulating hidden symmetries in graphene nanoribbons. Phys. Rev. Lett..

[54] Cai J., Ruffieux P., Jaafar R., Bieri M., Braun T., Blankenburg S., Muoth M., Seitsonen A. P., Saleh M., Feng X., Müllen K., Fasel R. (2010). Atomically precise bottom-up fabrication
of graphene nanoribbons. Nature.

[55] Son Y.-W., Cohen M. L., Louie S. G. (2006). Energy
gaps in graphene nanoribbons. Phys. Rev. Lett..

[56] Frisch, M. J. , Gaussian Inc 16 Revision C.01. Gaussian Inc.: 2016.

[57] Lu T., Chen F. (2012). Multiwfn: A multifunctional
wavefunction analyzer. J. Comput. Chem..

[58] Collier T. P., Saroka V. A., Portnoi M. E. (2017). Tuning
terahertz transitions in a
double-gated quantum ring. Phys. Rev. B.

[59] Song S., Teng Y., Tang W., Xu Z., He Y., Ruan J., Kojima T., Hu W., Giessibl F. J., Sakaguchi H., Louie S. G., Lu J. (2025). Janus graphene
nanoribbons
with localized states on a single zigzag edge. Nature.

[60] Cai J., Pignedoli C. A., Talirz L., Ruffieux P., Söde H., Liang L., Meunier V., Berger R., Li R., Feng X., Müllen K., Fasel R. (2014). Graphene nanoribbon
heterojunctions. Nat. Nanotechnol..

[61] Friedrich N., Brandimarte P., Li J., Saito S., Yamaguchi S., Pozo I., Peña D., Frederiksen T., Garcia-Lekue A., Sánchez-Portal D., Pascual J. I. (2020). Magnetism
of topological boundary states induced by boron substitution in graphene
nanoribbons. Phys. Rev. Lett..

[62] Blackwell R. E., Zhao F., Brooks E., Zhu J., Piskun I., Wang S., Delgado A., Lee Y.-L., Louie S. G., Fischer F. R. (2021). Spin splitting of dopant edge state
in magnetic zigzag
graphene nanoribbons. Nature.

[63] Wen E. C. H., Jacobse P. H., Jiang J., Wang Z., Louie S. G., Crommie M. F., Fischer F. R. (2023). Fermi-Level
engineering of nitrogen
core-doped armchair graphene nanoribbons. J.
Am. Chem. Soc..

[64] Jacobse P. H., Pizzochero M., Wen E. C. H., Barin G. B., Li X., Mutlu Z., Müllen K., Kaxiras E., Crommie M. F., Fischer F. R. (2025). Coupling of nondegenerate topological modes in nitrogen
core-doped graphene nanoribbons. ACS Nano.

[65] Chen L. (2017). Oriented graphene nanoribbons
embedded in hexagonal boron nitride
trenches. Nat. Commun..

[66] Taylor J., Guo H., Wang J. (2001). Ab initio modeling of quantum transport properties
of molecular electronic devices. Phys. Rev.
B.

[67] Taylor J., Guo H., Wang J. (2001). Ab initio
modeling of open systems: Charge transfer,
electron conduction, and molecular switching of a C_60_ device. Phys. Rev. B.

[68] Ke S.-H., Baranger H. U., Yang W. (2004). Electron transport through molecules:
Self-consistent and non-self-consistent approaches. Phys. Rev. B.

[69] Tamersit K. (2019). An ultra-sensitive
gas nanosensor based on asymmetric dual-gate graphene nanoribbon field-effect
transistor: proposal and investigation. J. Comput.
Electron..

[70] Arrhenius S. (1889). Über
die dissociationswärme und den einfluss der temperatur auf
den dissociationsgrad der elektrolyte. Z. Phys.
Chem..

[71] Parey V., Abraham B. M., Gaur N. K., Thapa R. (2022). First-principles study
of two-dimensional B-doped carbon nanostructures for toxic phosgene
gas detection. ACS Appl. Nano Mater..

[72] Peng S., Cho K., Qi P., Dai H. (2004). Ab initio study of CNT NO_2_ gas sensor. Chem. Phys. Lett..

[73] Jin Z., Wang C., Wu L., Song H., Yao X., Liu J., Zhao J., Zeng Z., Wang F. (2023). Fast responding and
recovering of NO_2_ sensors based on Ni-doped In_2_O_3_ nanoparticles. Sens. Actuators
B: Chem..

[74] Klaassen D. J., Eek L., Rudenko A. N., van’t Westende E. D., Castenmiller C., Zhang Z., De Boeij P. L., Van Houselt A., Ezawa M., Zandvliet H. J. W., Morais Smith C., Bampoulis P. (2025). Realization of a one-dimensional topological insulator
in ultrathin germanene nanoribbons. Nat. Commun..

[75] Plotnik Y., Rechtsman M. C., Song D., Heinrich M., Zeuner J. M., Nolte S., Lumer Y., Malkova N., Xu J., Szameit A., Chen Z., Segev M. (2014). Observation of unconventional
edge states in ‘photonic graphene’. Nat. Mater..

[76] Serra-Garcia M., Peri V., Süsstrunk R., Bilal O. R., Larsen T., Villanueva L. G., Huber S. D. (2018). Observation of a phononic quadrupole
topological insulator. Nature.

[77] Peri V., Serra-Garcia M., Ilan R., Huber S. D. (2019). Axial-field-induced
chiral channels in an acoustic Weyl system. Nat. Phys..

[78] Xia S., Liang Y., Tang L., Song D., Xu J., Chen Z. (2023). Photonic realization
of a generic type of graphene edge states exhibiting
topological flat band. Phys. Rev. Lett..

