# Supplementary material for: Topological Junction States in Graphene Nanoribbons: A Route to Topological Chemistry
Source: Nano Lett. 2025 Jun 18;25(26):10594–602. doi: 10.1021/acs.nanolett.5c02355 (PMC12232391; doi:10.1021/acs.nanolett.5c02355)
Supplement: Supplementary file 1 [file nl5c02355_si_001.pdf]

Supporting Information for
Topological junction states in graphene nanoribbons: A route to topological chemistry

Hazem Abdelsalam,[*,†,‡] Domenico Corona,[*,¶] Renebeth B. Payod,[*,§] Mahmoud A. S. Sakr,[*,‖] Omar H. Abd-Elkader,[*,⊥] Qinfang Zhang,[*,†] and Vasil A. Saroka[*,¶,#,@]

†*School of Materials Science and Engineering, Yancheng Institute of Technology, Yancheng 224051, P.R. China*

‡*Theoretical Physics Department, National Research Centre, Giza12622 Dokki, Egypt*

¶*Department of Physics, University of Rome Tor Vergata and INFN, Via della Ricerca Scientifica 1, 00133 Roma, Italy*

§*Institute of Physics, University of the Philippines, College, Los Baños, Laguna 4031 Philippines*

‖*Chemistry Department, Center of Basic Science, Misr University for Science and Technology (MUST), P.O. 77, Giza, Egypt*

⊥*Department of Physics and Astronomy, College of Science, King Saud University, P.O. Box 2455, Riyadh 11451, Saudi Arabia*

#*TBpack Ltd., 27 Old Gloucester Street, London, WC1N 3AX, United Kingdom*

@*Institute for Nuclear Problems, Belarusian State University, Bobruiskaya 11, 220030 Minsk, Belarus*

E-mail: hazemabdelhameed@gmail.com; domenico.corona@roma2.infn.it; rbpayod@up.edu.ph; mahmoud.sakr@must.edu.eg; omabdelkader7@ksu.edu.sa; qfangzhang@gmail.com; vasil.saroka@roma2.infn.it

Supporting Note 1: Methods

Density functional theory electronic structure calculations

The B3LYP hybrid functional,[1,2] combined with the 6-31G basis set,[3,4] is chosen for modeling due to its efficiency and accuracy with respect to the electronic properties of carbon-based materials.[5–7] Grimme-D3 Van der Waals correction[8] is applied to the B3LYP functional to account for long-range interactions with the target NO_2 molecule.

Quantum transport calculations

The local density approximation (LDA) is used for the exchange-correlation functional with an energy cutoff of 160 Ry. The reciprocal space k-grids are set to $1 \times 1 \times 100$ for the leads and $1 \times 1 \times 1$ for the scattering region. The current passing through the system is determined by Landauer-Büttiker formalism.[9] Thus, we sum the transmission probabilities, $T = \sum_i T_i$, for electron states, i, from one electrode to the other within the energy window subjected to the applied voltage V:

$$I = \frac{2e}{h} T \left(\mu_L - \mu_R\right) \, , \tag{S1}$$

where $\mu_L - \mu_R = |eV|$, and the μ_L and μ_R are the electrochemical potentials of the left and right electrodes, respectively.

Supporting Note 2: Electronic and topological properties of the N-dopped leads

The N-doping is a typical way of modifying the properties of carbon nanostructures.[10–13] Another possible alternative is a boron atom. The N and B atoms are frequently used because they can form hexagonal boron nitride having a honeycomb lattice that is compatible with that of graphene due to approximately equal lattice constants. In carbon nanostructures

nitrogen is used for n-doping (electrons), while boron is used for p-doping (holes). This is what is basically seen in the band structure plots for our N-doped leads based on AGNR(7) topological unit cell in Figure S1.

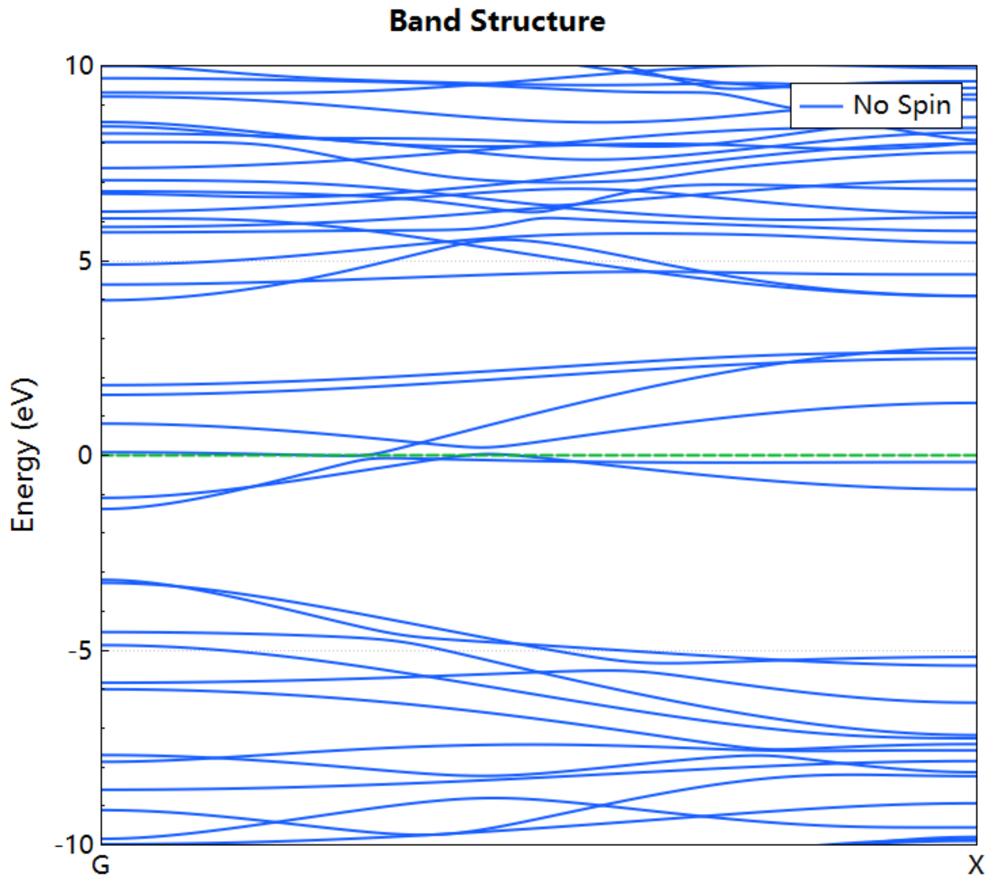

Figure S1. Electronic energy bands structure of N-dopped leads of the NO_2 sensor proposed in Fig. 4 of the main text. Dashed green line is the Fermi level.

In some cases, substitutional doping can affect the topological properties of the GNRs. The effect of changing a topological order by using B-doping was predicted in the seminal paper on GNR classification.[14] Several recent studies, which focus on N-doping, report similar changes.[12,13] However, these studies deal with a dilute doping and a configuration that features mirror-symmetric positioning of the dopants with respect to the translation axis of the ribbon. In contrast, our N-doping configuration breaks the mirror symmetry of the pristine AGNR(7) along the translation axis. Broken mirror symmetry prevents quantization of the Zak phase and makes the Z_2 invariant ill-defined in the sense that the invariant is

not an integer anymore, even though it can still be formally calculated via Eq. (2) of the main text by projecting wave functions onto the occupied states of the pristine ribbon. Note that, as a result of doping, the actual Fermi level is in the conduction band, thereby making the structure metallic. For metallic structures, the Z_2 invariant does not make sense as we explained in the manuscript.

In Figure S2, we model N-doping in the tight-binding model simultaneously tracking the Z_2 invariant obtained by projection onto the occupied states below the pristine ribbon gap. For this, we set non-zero on-site energies for the N-atoms and varied the strength of the hopping integrals for N-C bonds with respect to the normal C-C bonds. The deviation from the integer value of the Z_2 invariant comes from the on-site energies. In Figure S2, N atoms are red, and their sizes reflect on-site energies $t_{0,\mathrm{N}}$, while N-C bonds are cyan, and their width reflects the strength of the hopping integral $t_{1,\mathrm{CN}}$. For clarity, the N-atoms and N-C bonds are also contoured by persistent black circles and lines, respectively. The yellow line is the position of the actual Fermi level that is different from zero because of the 4 additional electrons per unit cell supplied by the N atoms. When the yellow line is not seen, it coincides with the upper flat band. It is seen from the modeling that the N-doped leads are metallic and their topological properties are irrelevant to the given performance.

References

(1) Becke, A. D. Density-functional thermochemistry. III. The role of exact exchange. *J. Chem. Phys.* **1993**, *98*, 5648–5652.

(2) Lee, C.; Yang, W.; Parr, R. G. Development of the Colle-Salvetti correlation-energy formula into a functional of the electron density. *Phys. Rev. B* **1988**, *37*, 785–789.

(3) Ditchfield, R.; Hehre, W. J.; Pople, J. A. Self-consistent molecular-orbital methods. IX. An extended Gaussian-type basis for molecular-orbital studies of organic molecules. *J. Chem. Phys.* **1971**, *54*, 724–728.

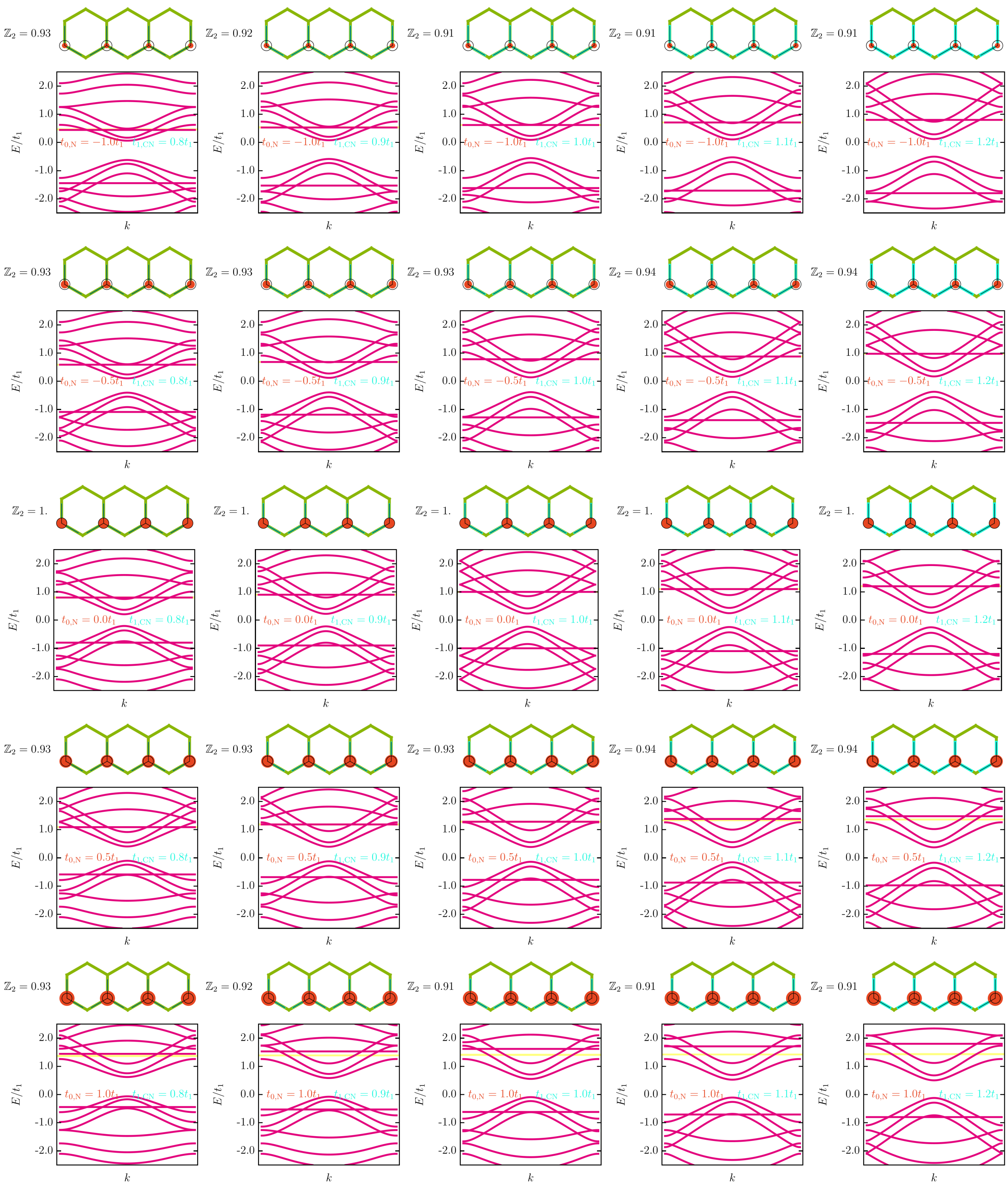

Figure S2. The tight-binding energy bands of N-dopped leads for the varying N atom on-site energies (top-to-bottom) and NC bond hopping integrals (left-to-right). Yellow line is the Fermi level. Each panel is supplemented by a graphical highlight of the used parameters within a unit cell of the ribbon [implying the Oy-axis as a translation one]. The $Z_2 \equiv \mathbb{Z}_2$ is displayed to the left of the unit cell.

(4) Hehre, W. J.; Ditchfield, R.; Pople, J. A. Self—consistent molecular orbital methods. XII. Further extensions of Gaussian—type basis sets for use in molecular orbital studies of organic molecules. *J. Chem. Phys.* **1972**, *56*, 2257–2261.

(5) Povie, G.; Segawa, Y.; Nishihara, T.; Miyauchi, Y.; Itami, K. Synthesis and size-dependent properties of [12], [16], and [24] carbon nanobelts. *J. Am. Chem. Soc.* **2018**, *140*, 10054–10059.

(6) Abdelsalam, H.; Sakr, M. A.; Saroka, V. A.; Abd-Elkader, O. H.; Zhang, Q. Nanoporous graphene quantum dots constructed from nanoribbon superlattices with controllable pore morphology and size for wastewater treatment. *Surf. Interfaces* **2023**, *40*, 103109.

(7) Abdelsalam, H.; Abd-Elkader, O. H.; Sakr, M. A. S.; Saroka, V. A.; Zhang, Q. Nanoporous triangulene-based frameworks for the separation of petroleum hydrocarbons: Electronic, magnetic, optical, and adsorption properties. *ACS Appl. Nano Mater.* **2023**, *6*, 15128–15137.

(8) Grimme, S.; Antony, J.; Ehrlich, S.; Krieg, H. A consistent and accurate *ab initio* parametrization of density functional dispersion correction (DFT-D) for the 94 elements H-Pu. *J. Chem. Phys.* **2010**, *132*, 154104.

(9) Datta, S. In *Electronic transport in mesoscopic systems*; Haroon, A., Pepper, M., Broers, A., Eds.; Cambrige University Press: Cambridge, United Kingdom, 1995.

(10) Cai, J.; Pignedoli, C. A.; Talirz, L.; Ruffieux, P.; Söde, H.; Liang, L.; Meunier, V.; Berger, R.; Li, R.; Feng, X.; Müllen, K.; Fasel, R. Graphene nanoribbon heterojunctions. *Nat. Nanotechnol.* **2014**, *9*, 896–900.

(11) Friedrich, N.; Brandimarte, P.; Li, J.; Saito, S.; Yamaguchi, S.; Pozo, I.; Peña, D.; Frederiksen, T.; Garcia-Lekue, A.; Sánchez-Portal, D.; Pascual, J. I. Magnetism of topological boundary states induced by boron substitution in graphene nanoribbons. *Phys. Rev. Lett.* **2020**, *125*, 146801.

(12) Wen, E. C. H.; Jacobse, P. H.; Jiang, J.; Wang, Z.; Louie, S. G.; Crommie, M. F.; Fischer, F. R. Fermi-Level engineering of nitrogen core-doped armchair graphene nanoribbons. *J. Am. Chem. Soc.* **2023**, *145*, 19338–19346.

(13) Jacobse, P. H.; Pizzochero, M.; Wen, E. C. H.; Barin, G. B.; Li, X.; Mutlu, Z.; Müllen, K.; Kaxiras, E.; Crommie, M. F.; Fischer, F. R. Coupling of nondegenerate topological modes in nitrogen core-doped graphene nanoribbons. *ACS Nano* **2025**, *19*, 13029–13036.

(14) Cao, T.; Zhao, F.; Louie, S. G. Topological phases in graphene nanoribbons: Junction States, spin centers, and quantum spin shains. *Phys. Rev. Lett.* **2017**, *119*, 076401.
